# Address by the President, Dr. W. H. Todd, D.D.S.

**Published:** 1896-01

**Authors:** 


### Address by the President, Dr. W. H. Todd, D.D.S.

Read before the Ohio State Dental Society, December 3d, 1895.

Members of the Ohio State Dental Society: It has been customary for your President to make an annual address. There would be nothing more appropriate than to make some general observations concerning the responsibilities centered upon this as State Society, the necessity of an increase of membership, and the influence it would have on the community at large if that could be brought about.

It must be borne in mind that this, as a State Society, has a responsibility that is not accorded to any local District Society. We are expected to formulate and promote any State law pertaining to dentistry, and if that law should lack any of the essentials necessary, even if it should be formulated by some interest foreign to the State Society, we are held accountable.

We should always take that dignified, unselfish and self-sacrificing professional attitude, which the general public so little understands and appreciate, knowing that our responsibility to the human family is fully as great, even if, through ignorance, they try to open the door of legislation so wide that dental legislation may itself be prostituted to purposes of dishonest jobbery and quackery.

Again, as a State Society, we are looked upon as representing the advanced thinkers and original investigators of dentistry in our State. As such we should make a prior claim to all original matter, and in return we can encourage these workers with our thoughtful sympathy and counsel, taking care that their efforts, with the discussions that take place, will be carefully reported and placed before the profession.

There are dental journals throughout the State that should foster all interests in the Society and delegate themselves its official organs. In that way an interest could be encouraged in localities where there is none, thereby benefiting both the community and our Society.

Among the many perplexing questions which come before us is, how to bring up the standard of the dental profession. The college faculty is doing a noble work in the opportunities it gives a student and the high grade it requires to graduate, but this faculty can look around and see some of its best students stooping to the small and mean ways in the practice of dentistry that are so condemned by the profession.

Now, the question arises, what shall we do with the worthy graduate? Let him shift for himself? He has passed all the chairs and received his diploma, giving him the right to practice dentistry. He may have impressed all with his fitness for his profession, not alone by his desire to be the best in his class, but by the sense of duty that compels him to perfect himself in his chosen profession, so that his services may be truly worth their hire.

There are forty-eight dental schools in this country, with 1,208 graduates for 1895. How many of that number have joined our Societies?

The responsibility of the college faculty does not cease on the day the student graduates, for the diploma he takes is the faculty's endorsement of his professional standing and ability. Dr. J. Y. Crawford, in his address before the American Dental Association, requested that all reputable dental colleges formulate a uniform oath or obligation to which the student should subscribe. The faculty should impress the student with the importance of joining dental societies. He could then be made to feel that he had found a home where he could mature into a full-fledged member of a progressive profession where his mind, which has been so carefully trained, will find a field in which to expand and grow, and where he can become an original investigator in some of the many branches of the profession; whereas, if he be left to himself, he may be attracted by the many advertisements which are to be seen, and begin to lower the standard and character of dentistry by producing cheap and poor work, with a reckless use of some nostrum, of the contents of which he knows nothing, regardless of consequences for the sake of deceiving the public and gaining a fee—having but one desire, that to make money—having but one aim, that of doing cheap work at a high price.

With 25,000 dentists, what portion of that number attend our meetings?

If the Ohio State Dental Society be a representative the number is very small.

The dentist who fails to read the dental literature of to-day becomes a back number. The dentist who does not attend at least one meeting of a dental society during the year loses all interest in the elevation of his profession, and what right would he have to censure the layman for disrespect if he shows such a lack of interest.

If each member would exert himself and persuade one or more dentists from his locality to attend these meetings, with the increased number, what a power we might be and what an influence we might have in the community.

In concluding, I wish to express my pleasure at meeting with you again this year, that we may not only talk over some of the new thoughts that are to be presented at this meeting, but congratulate each other and the committee on the splendid representation we had from Ohio at the tri-state meeting.

We might consider here the invitation which was extended and accepted by the State Dental Association of Michigan and Indiana to meet with us in 1898, for we should all appreciate the work connected with such a meeting and do everything possible to assist the committee in their arrangements.

Since our last meeting death has taken from our midst a worthy and talented member of this Society, Dr. W. H. Sedgwick, sr., of Granville, Ohio, and who was President in 1890.

